# Follicular Helper T Cells (Tfh) and IL-21 Involvement in the Pathogenesis of Bullous Pemphigoid

**DOI:** 10.1371/journal.pone.0068145

**Published:** 2013-07-05

**Authors:** Qiuju Li, Zhenfeng Liu, Erle Dang, Liang Jin, Zheng He, Luting Yang, Xiaowei Shi, Gang Wang

**Affiliations:** Department of Dermatology, Xijing Hospital, Fourth Military Medical University, Xi’an, People’s Republic of China; UTHealth Medical School, United States of America

## Abstract

The pathogenesis of bullous pemphigoid (BP) is characterized by the T cell-dependent production of autoantibodies. Recent studies have indicated that follicular T helper cells (Tfh), the key modulator of B cell activation and autoantibody production, are critical in the development of several autoimmune diseases. Tfh cells perform their functions via IL-21, their hallmark cytokine. In the present study, the frequencies of Tfh cells were investigated in the peripheral blood samples of BP patients to evaluate whether Tfh cells involve in this clinical entity. Significantly higher Tfh cell counts were observed in the peripheral blood of BP patients than those in healthy controls (median: 11.25% vs. 4.95%, respectively; *P<*0.001). Additionally, the serum IL-21 levels in BP patients were higher than those of the healthy controls (median: 103.98 pg/mL vs 46.77 pg/mL, respectively; *P<*0.001). The frequencies of Tfh cells and IL-21 levels were both positively correlated with anti-BP180-NC16A autoantibody titers (*R = *0.712, *P<*0.01 and *R = *0.578, *P = *0.030, respectively). After effective therapy, the frequencies of Tfh cells as well as the serum IL-21 levels in BP patients decreased along with clinical improvement. Most importantly, Tfh depleted CD4^+^ T cells and anti-IL-21 neutralization antibody could inhibit the T cell-induced B cell activation and secretion of BP autoantibody *in vitro*. Those results suggest that Tfh cells play an important role in autoantibody production and are involved in the pathogenesis of BP.

## Introduction

Bullous pemphigoid (BP) is an autoimmune subepidermal blistering disease characterized by production of autoantibody directly responding to hemidesmosomal proteins within the dermal-epidermal junction [1], [2], [3]. The production of autoantibodies directed against the non-collagenous 16A domain (NC16A), the transmembrane domain of the hemidesmosomal protein (BP180), was the initiating event of the pathomechanism [4], [5], [6], [7], [8]. Titers of circulating anti-BP180-NC16A autoantibodies were considered to be clinical severity markers that reflected the severity and activity of the disease [4], [9], [10]. However, the source of the autoantibodies and the mechanism underlying their emergence remain unclear.

Follicular T helper cells (Tfh) have recently emerged as a separate subset of CD4^+^ T helper cells [11]. The major function of Tfh cells is to help B cells activation and antibody production during humoral immune responses, specifically via interactions between molecules on the surface of Tfh cells and receptors or ligands located on the surface of B cells [12]. Consist with the location in B cell follicles, Tfh cells express high levels of CXCR5, ICOS, PD-1, CD40 ligand (CD40L), OX40, and SLAM-associated protein (SAP), allowing themselves to migrate to the germinal center (GC) and then to activate B cells [13], [14]. IL-21 was a cytokine preferentially expressed by Tfh cells and served as an important regulator of humoral responses by directly regulating B-cell proliferation and class switching [15], [16], [17]. It has been reported that human circulating Tfh cells were in proportion to their GC counterparts [18]. Abnormal Tfh cells frequency and certain molecules highly expressed by Tfh cells have been observed in mice and humans with autoimmune diseases [19]. However, little is currently known about the potential role of Tfh cells in autoimmune blistering disease.

The present study aimed to determine whether Tfh cells play an important role in pathogenic autoantibody production in BP and to clarify their involvement in the pathogenesis of BP. The increased frequency of Tfh cells and level of IL-21 were detected in BP patients, which were positively correlated with high level of serum anti-BP180-NC16A autoantibody. In addition, we found that Tfh cells and IL-21 were the essential part for the secretion of anti-BP180-NC16A in T cell/B cell co-culture system *in vitro*. Thus, these results have indicated the possible involvement of Tfh cells and IL-21 in the pathogenesis of BP.

## Results

### Patient Characteristics

Overall, 32 patients with active BP were involved in the study. The characteristics of the patients are shown in **Table S1**. We collected serum samples from 14 BP patients (no. 01–14; age range: 45–77 years) and 14 sex- and age-matched healthy controls. The ages of the patients and the healthy controls were not significantly different (63.36±9.62 years vs. 58.93±6.21 years, respectively; *P = *0.162). Peripheral blood mononuclear cells (PBMCs) were isolated from 20 BP patients (no. 07–26; age range: 32–89 years) and 20 sex- and age-matched healthy controls. The ages of the patients and the healthy controls were not significantly different (63.20±15.98 years vs. 58.65±12.31 years, respectively; *P = *0.320). CD19^+^ B cells and CD4^+^ T cells were isolated from 6 BP patients (no. 27–32; age range: 44–80 years) and 6 sex- and age-matched healthy controls. The ages of the patients and the healthy controls were not significantly different (62.17±15.17 years vs. 59.17±10.15 years, respectively; *P = *0.398).

### Increased Serum IL-21 Levels and the Positive Correlation between IL-21 Levels and anti-BP180-NC16A Autoantibody Titers in BP Patients

**Figure 1:**
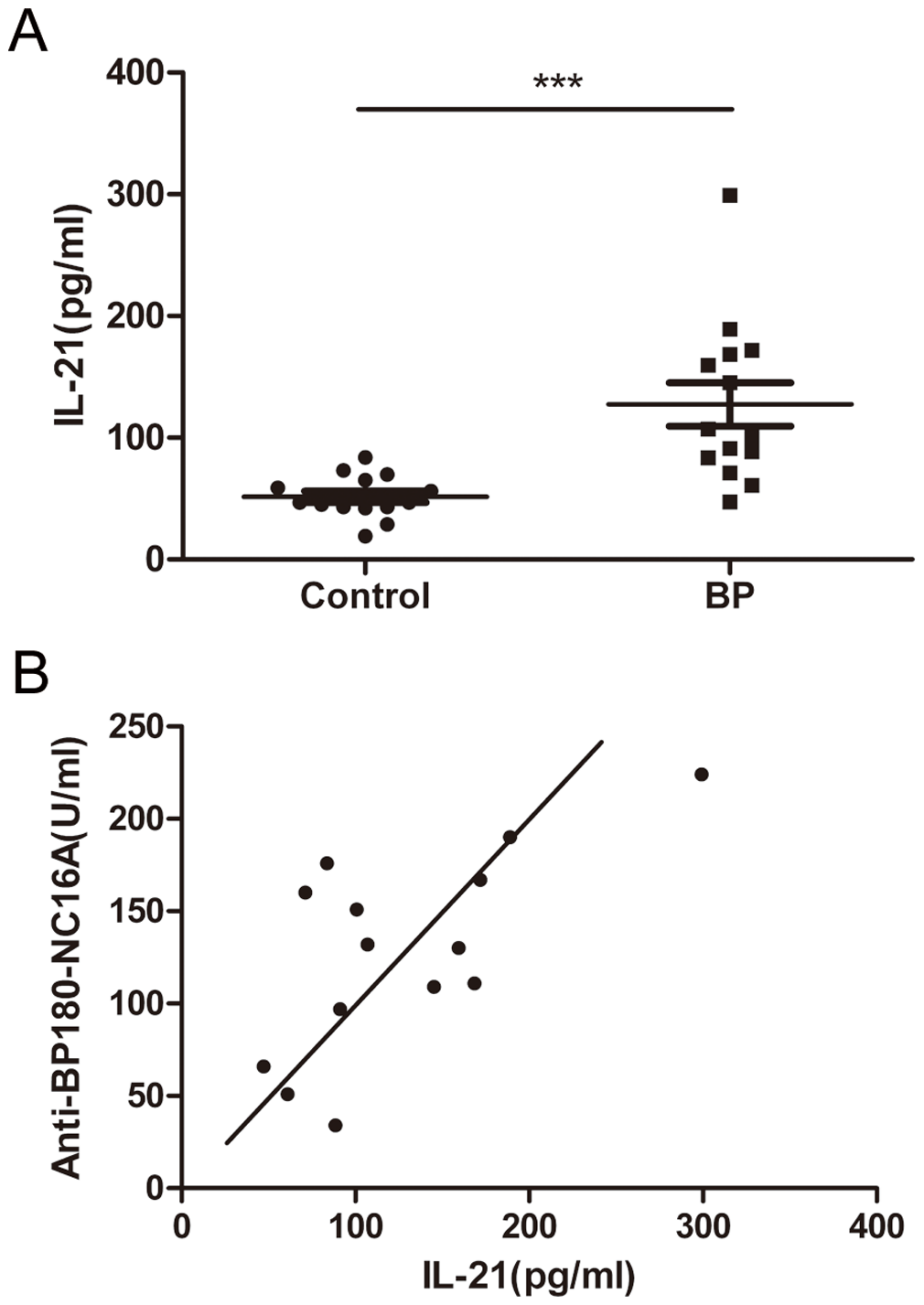
IL-21 levels in the sera of BP patients and healthy controls. (**A**) IL-21 levels in the sera of 14 BP patients and 14 age-matched and sex-matched healthy controls. Each data point represents an individual subject; horizontal lines show the 25^th^ percentile, the median, and the 75^th^ percentile. (**B**) The correlation between the IL-21 levels and the anti-BP180-NC16A autoantibody titers of 14 BP patients. Each data point represents an individual subject. ***P<0.001.

It becomes clear that IL-21 produced by Tfh cells serve as an important regulator of humoral responses. We detected the IL-21 levels in the sera of 14 BP patients (no. 01–14) and 14 age- and sex-matched healthy controls by ELISA. There was a significant increase in IL-21 levels in BP patients as compared with those in the healthy controls (median: 103.98 pg/mL vs. 46.77 pg/mL, respectively; *P*<0.001) (Fig. 1A). Then, we determined the anti-BP180-NC16A autoantibody titers in BP patients and analyzed the correlation between the autoantibody titers and the IL-21 levels in the sera of the BP patients. The serum IL-21 levels were positively correlated with the anti-BP180-NC16A autoantibody titers in BP patients (*R = *0.578, *P = *0.030) (Fig. 1B).

### Increased Tfh cells and the Positive Correlation between Tfh cell Populations and anti-BP180-NC16A Autoantibody Titers in BP Patients

**Figure 2:**
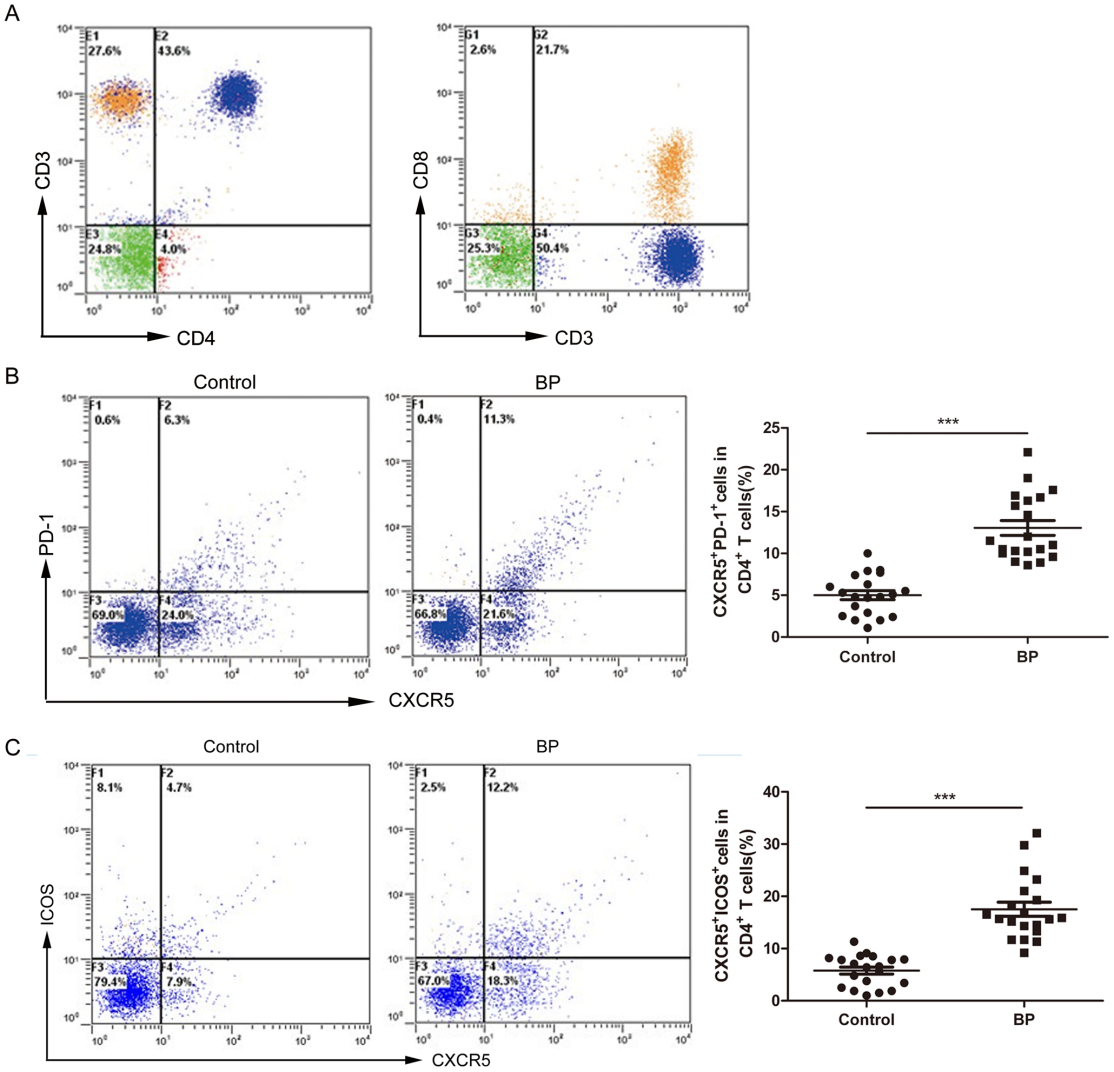
The proportion of Tfh cells in the peripheral blood of BP patients. (**A**) Upper right quadrant: percentages of CD3^+^CD4^+^ and CD3^+^CD8^+^ cells (43.6% and 21.7%, respectively); these agreed with previous results. (**B**) The percentages of CXCR5^+^PD-1^+^ cells among the CD4^+^ T lymphocytes of 20 BP patients and 20 healthy controls. (**C**) The percentages of CXCR5^+^ICOS^+^ cells among the CD4^+^ T lymphocytes of 20 BP patients and 20 healthy controls. Each data point represents an individual subject. Each data point represents an individual subject. ***P<0.001.

We collected blood samples from 20 BP patients (no. 07–26) and 20 age- and sex-matched healthy controls into EDTA tubes and isolated the PBMCs. The percentages of Tfh cells in the peripheral blood of the BP patients and healthy controls were detected and analyzed by flow cytometry (FCM). The percentages of CXCR5^+^PD-1^+^ and CXCR5^+^ICOS^+^ cells among the CD4^+^ T lymphocytes were used to represent the circulating Tfh cells [12], [20], [21]. There was a significant increase in the percentage of CXCR5^+^PD-1^+^ cells among the CD4^+^ T lymphocytes in the BP patients as compared with those in the healthy controls (median: 11.25% vs. 4.95%, respectively; *P<*0.001) (Fig. 2A, B). Moreover, the percentages of CXCR5^+^ICOS^+^ cells among the CD4^+^ T lymphocytes in BP patients were also higher than those in the healthy controls (median: 15.80% vs. 6.50%, respectively; *P<*0.001) (Fig. 2C). These results suggested that circulating Tfh cells were increased in BP patient.

**Figure 3:**
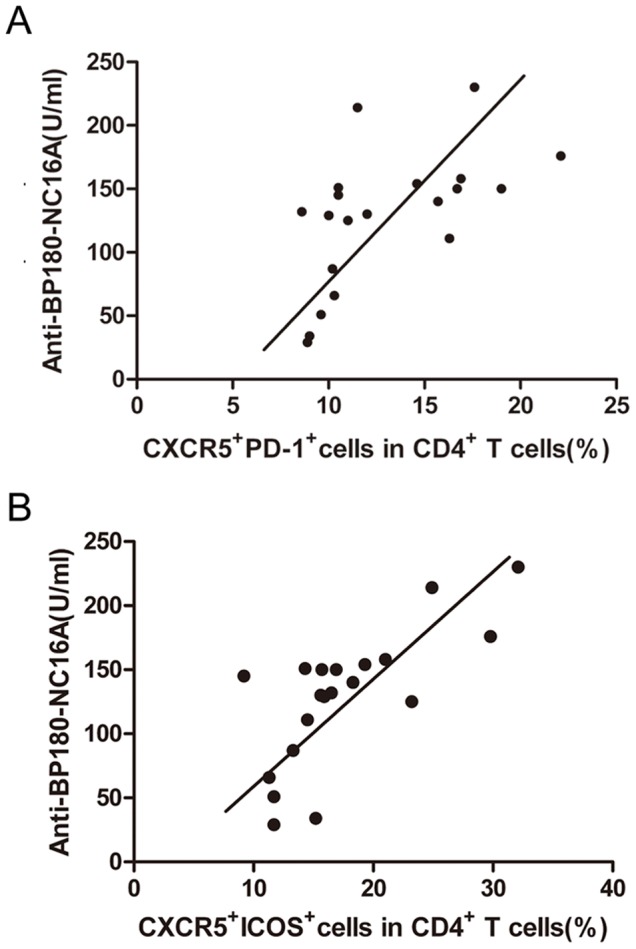
The correlation between the percentage of Tfh cells and the anti-BP180-NC16A autoantibody titers in the peripheral blood of BP patients. (**A**) The correlation between the percentages of CXCR5^+^PD-1^+^ cells among the CD4^+^ T lymphocytes and the anti-BP180-NC16A autoantibody titers of 20 BP patients. (**B**) The correlation between the percentage of CXCR5^+^ICOS^+^ cells among the CD4^+^ T lymphocytes and the anti-BP180-NC16A autoantibody titers of 20 BP patients. Each data point represents an individual subject.

We then analyzed the correlation between the anti-BP180-NC16A autoantibody titers and the percentages of Tfh cells. The percentages of CXCR5^+^PD-1^+^ cells among CD4^+^ T lymphocytes showed a positive correlation with the anti-BP180-NC16A autoantibody titers (*R = *0.712, *P<*0.01) (Fig. 3A). The correlation between the percentages of CXCR5^+^ICOS^+^ cells among CD4^+^ T lymphocytes and the anti-BP180-NC16A autoantibody titers was also analyzed, and a similarly positive correlation was observed (*R = *0.708, *P<*0.01) (Fig. 3B).

### Successful Therapy Decreases IL-21 Levels and Tfh cell Populations in BP Patients

**Figure 4:**
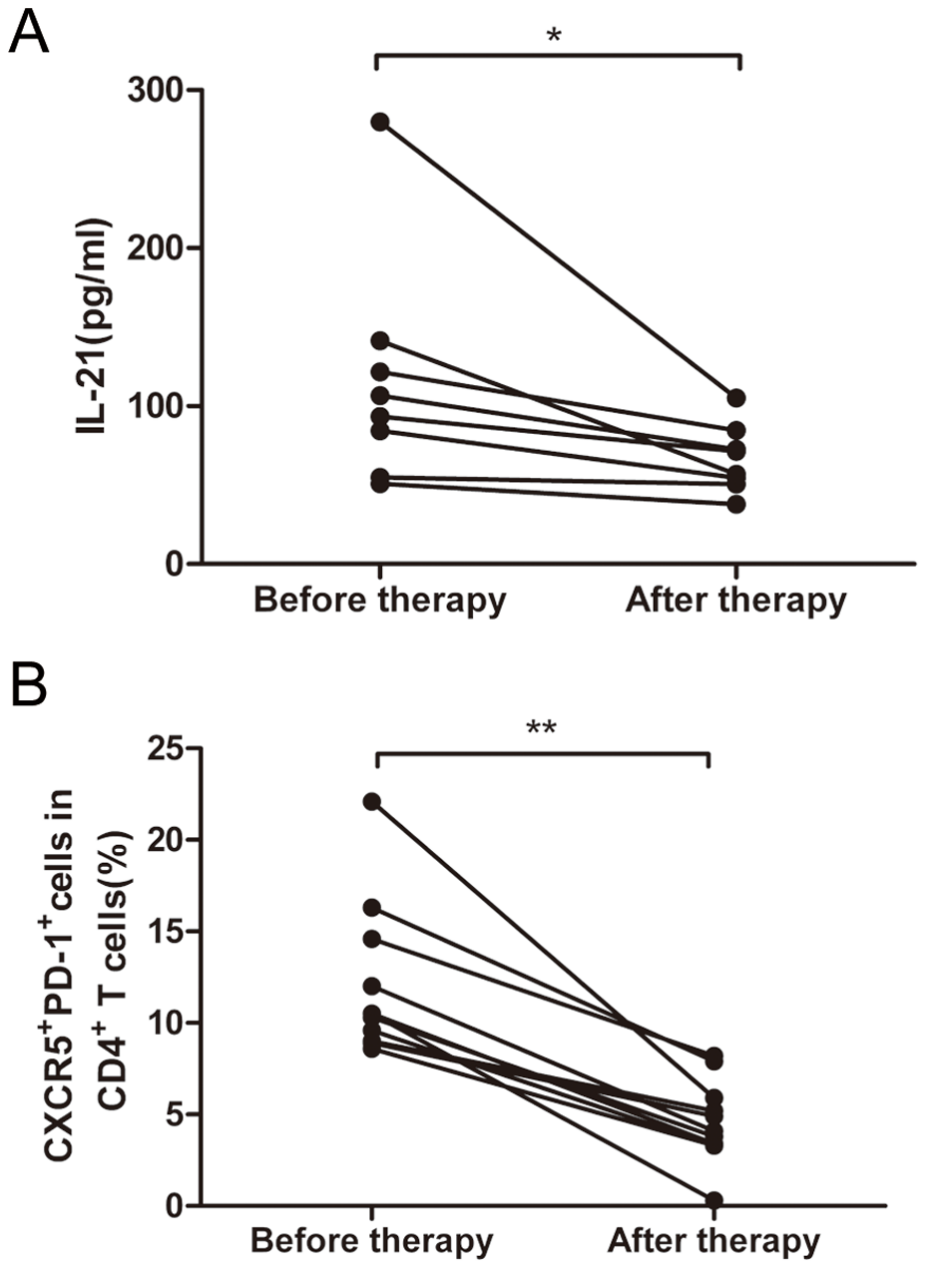
The IL-21 levels and Tfh cells in BP patients before and after therapy. (**A**) The IL-21 levels of 8 BP patients before and after effective and normative therapy. According to the severity of disease and the whole healty condition, the patients were geiven methylprednisolone for venous transfusion or oral administration and some drugs for external use to prevent infection and promote skin healing. Each data point represents an individual subject before therapy (left) or after therapy (right). (**B**) The percentage of CXCR5^+^PD-1^+^ cells among the CD4^+^ T lymphocytes of 11 BP patients before and after effective and normative therapy. Each data point represents an individual subject before therapy (left) or after therapy (right). *P<0.05, **P<0.01.

According to the severity of disease and the whole healthy condition, the patients were given methylprednisolone for venous transfusion or oral administration, and some drugs for external use to prevent infection and promote skin healing. We then collected the serum samples from 8 BP patients (no. 01–08) with symptomatic improvement and the scores were 0. The serum IL-21 levels of BP patients were measured before and after therapy using ELISA. The serum IL-21 levels after therapy were lower than those before therapy (median: 99.98 pg/mL vs. 64.08 pg/mL, respectively; *P = *0.012) (Fig. 4A).

Similarly, PBMCs of 11 BP patients (no. 07–17) were obtained at regular intervals following symptomatic improvement with effective therapy. The percentages of CXCR5^+^PD-1^+^ cells among the CD4^+^ T lymphocytes were determined after therapy. A highly significant reduction in the percentage of CXCR5^+^PD-1^+^ cells among CD4^+^ T lymphocytes was observed after therapy as compared to those before therapy (median: 10.50% vs. 4.10%, respectively; *P = *0.003) (Fig. 4B).

### Depletion of CD4^+^CXCR5^+^ T cells or Blockade of IL-21 inhibit T cell-induced Autoantibody Secretion

**Figure 5:**
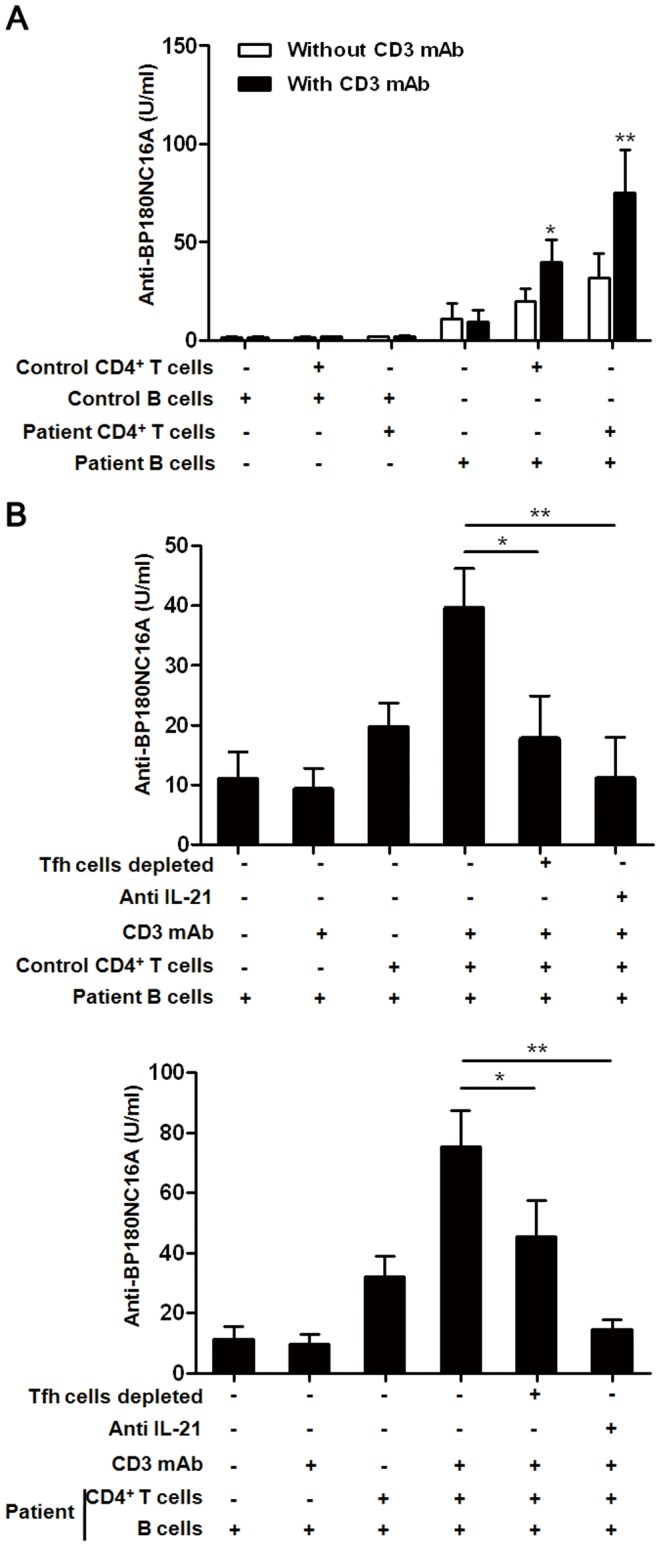
Tfh cells help the secretion of autoantibody in CD4^+^ T cells/B cells co-cuture *in vitro*. (**A**) Secretion of autoantibody in CD4^+^ T cells/B cells co-cuture system. Peripheral CD19^+^ B cells(1.5×10^5^ cells/well) and CD4^+^ T cells(1.5×10^5^ cells/well) from 6 healthy controls and 6 BP patients were cultured alone or together for 7 days and then the secretion of autoantibody were detected. (**B**) **S**ecretion of autoantibody in CD4^+^ T cells/B cells co-cuture system after depletion of Tfh or inhibition of IL-21. CD4^+^CXCR5^+^ T cells were cleaved from the co-culture system by sorting on a FACSAria flow cytometer to evaluate the function of Tfh cells in BP autoantibody secretion. The effect of inhibiting IL-21 were examined by adding neutralizing anti-IL-21 polyclonal Ab. CD19^+^ B cells alone or culture with anti-CD3 Ab were the negative control. Results shown are from a representative experiment of three independent experiments. *P<0.05, **P<0.01.

It has been previously reported that anti-CD3-activated human CD4^+^ T cell have been shown to induce Ig production from peripheral blood B cell. We now extend these results to demonstrate the function of activated CD4^+^CXCR5^+^ T cells subset in helping B cell to secret pathogenic autoantibody. CD19^+^ B cells and CD4^+^ T cells, isolated from 6 BP patients (no. 27–32) and 6 sex- and age-matched healthy controls, were cultured alone or together for 7 days and then the secretion of autoantibody were detected. The co-culture system showed that CD4^+^ T cells of healthy control or BP patient activated by immobilized anti-CD3 induce anti-BP180-NC16A autoantibody secretion from BP peripheral blood B cells (Fig. 5A). In contrast, healthy control B cells did not undergo efficient autoantibody secretion with or without CD3 mAb activated T cells from healthy control or BP patient. The depletion of CD4^+^CXCR5^+^ T cells can significantly decrease the secretion of anti-BP180-NC16A autoantibody (P<0.05) (Fig. 5B). To determine whether blockade of IL-21 modulated the secretion of pathogenic autoantibody, anti-IL-21 neutralizing Abs to were used in the co-culture system. The anti-BP180-NC16A autoantibody secretions were significantly inhibited by IL-21 blockade (P<0.05) (Fig. 5B). These results showed that Tfh cells and IL-21 play an important role in pathogenic autoantibody production in BP.

## Discussion

Tfh cells have received more attention in recent years because they were considered to be responsible for helping B cells during the antibody response [22]. They have been proved to be involved in several autoimmune diseases [23], [24], [25]. In this study, an increase in the Tfh cell counts was observed in the peripheral blood of BP patients as compared with those of healthy controls. Serum IL-21 level, which is the major cytokine secreted by Tfh cells, was also increased. The percentages of Tfh cells and the IL-21 levels were positively correlated with the anti-BP180-NC16A autoantibody titers in BP patients. This autoantibody titer has been recognized as a serum marker of the severity and the activity of the disease [9], [10]. More importantly, depletion of CD4**^+^**CXCR5**^+^** T cells can decreased the secretion of BP autoantibody in anti-CD3-activated human CD4^+^ T cells/B cells co-culture system, and so as to the blockade of IL-21 by neutralizing Ab. From the present results, we considered that Tfh cells and IL-21 play an important role in the production of pathogenic autoantibody are involved in the pathogenesis of BP.

As a separate subset of CD4^+^ T helper cells, Tfh cells localize in the B cell follicles; the relevant transcription factor for these cells is Bcl-6, which distinguishes them from Th1, Th2, and Th17 cells [14], [26]. These Tfh cells express high levels of the chemokine receptor CXCR5, which allows their chemotaxis and retention in the lymphoid follicle, where they can make contact with antigen-primed B cells, leading to B cell proliferation, isotype switching, and the somatic mutation of the Ig repertoire [11], [27], [28]. CXCR5, ICOS, CD40 L, and PD-1 are markers of Tfh cells [29], whereas IL-21 is the major cytokine secreted by Tfh cells. The important function of Tfh cells is to help B cells produce high-affinity antibodies via humoral immunity through the cognate Tfh-B cell interaction in the GC of B cell follicles in secondary lymphoid organs [19], [30]. Intense B cell competed for a small number of CD4^+^ T cells has been observed using intravital microscopy [31]. Moreover, the absence of T cell “help” during B cell priming leaded to B cell apoptosis rather than differentiation into GC B cells or plasma cells [32]. Therefore, abnormal Tfh cells may lead to the production of abnormal autoantibodies.

The production of high-affinity antibodies is the most important characteristic of many autoimmune diseases. Tfh cells likely provide inappropriate helper signals to self-reactive B cells in antibody-mediated autoimmune diseases [33]. In both mice and humans with autoimmune diseases, such as SLE, RA, MG, pSS, and NOD diabetes, strong evidences support the involvement of Tfh cells in autoantibody production and GC formation, either through aberrant Tfh cells or the abnormal but important molecules of Tfh cells [23], [24], [25], [34], [35], [36]. In numerous murine models of autoimmunity, the inhibition of the function of Tfh cell-associated molecules, such as CD40 L, ICOS, and IL-21, has been shown to result in reduced autoantibody production [12], [37], [38], [39]. Patients with SLE treated with CD40L-specific antibodies had fewer plasma cells that secrete DNA-specific antibodies, with this treatment ameliorating the disease pathology [19]. Linterman et al. established a novel pathogenic pathway in the sanroque mouse model of lupus, in this model, pathological GC occurrence was observed subsequent to an increased number of Tfh cells, leading to the production of high-affinity anti-double-stranded DNA antibodies, which eventually caused end-organ inflammation [13], [18]. This pathway was also confirmed in a subset of patients with systemic lupus erythematosus [27]. BP is a classic autoimmune disease, and abnormal Tfh cells were observed in BP patients in this study. Moreover, the measurement of the IL-21 levels and the percentages of Tfh cells in BP patients after effective and normative therapy, as indicated by symptomatic improvement, revealed that the levels of both indicators were decreased after therapy as compared to those before therapy. Meanwhile, depletion of Tfh cells or blockade of IL-21 can inhibit T cell-induced autoantibody secretion in CD4^+^ T cells/B cells coculture system *in vitro*. This provided further evidence that Tfh cells were involved in the pathogenesis of BP and that they may play a role in the production of pathogenic autoantibodies.

The surface phenotype of Tfh cells, particularly “circulating Tfh cells,” has been controversial owing to only a partial knowledge of Tfh cells. In our study, CD4, CXCR5, PD-1, and ICOS were chosen as markers for Tfh cells based on the latest studies [19], [27], [29], [40]. The proportions of CXCR5^+^PD-1^+^ and CXCR5^+^ICOS^+^ cells among CD4^+^ T lymphocytes were found to be increased in BP patients as compared with those in the healthy controls. Therefore, to a certain extent, CD4^+^CXCR5^+^PD-1^+^ and CD4^+^CXCR5^+^ICOS^+^ cells can both be considered representative of circulating Tfh cells.

BP is a typical example of an autoimmune blistering skin disease that is well known for its intractability and propensity for recurrent attacks [37], [38]. BP is a prototypical antibody-mediated, organ-specific autoimmune disease [39]. The binding of autoantibodies to antigens initiates a series of immune inflammatory events, including complement activation, mast cell degranulation, macrophage activation, and neutrophilic infiltration, which leads to the development of clinical blisters or bullae [39], [41]. The binding of the pathogenic autoantibody to a specific antigen is the initiating factor for BP. However, the source and the mechanism of autoantibody production remain unclear. Our findings may provide important clues for a further evaluation of the source of the autoantibody involved and the pathogenesis as well as more effective therapy of BP.

In conclusion, we assessed Tfh cell counts and IL-21 levels in order to explore whether Tfh cells play a role during the production of autoantibodies in BP patients. Our results suggest that Tfh cells participate in the pathogenesis of BP. Further studies are need to determine whether Tfh cells initiate autoantibody production in BP and to clarify the mechanisms involved.

## Materials and Methods

### Ethics Statement

Written informed consent was obtained from all the subjects. This study was approved by the local ethics committee of Xijing Hospital, The Fourth Military Medical University, Xi’an, China.

### Patients

We collected sera from 14 BP patients and PBMCs from 20 BP patients of either sex at the Department of Dermatology, Xijing Hospital, including inpatients and outpatients from November 2009 to November 2011. All subjects were newly diagnosed with BP based on the typical clinical and histological presentation, direct or indirect immunofluorescence examination, and the presence of circulating autoantibodies. None of the patients had received any form of immunosuppressive therapy at the time of inclusion in the study. A total of 34 normal controls matched with patients in terms of sex and age were chosen from the Medical Examination Center of Xijing Hospital. None of the controls presented any evidence of chronic autoimmune diseases or acute infections in their blood. According to the scored standard for the disease severity from Tsuji-Abe et al. [42], we scored every patient as follows: 0 point, no lesions (erythema, blisters, erosions); 1 point, ≤20% of skin involvement; 2 points, 21%–40% of skin involvement; 3 points, 41%–60% of skin involvement; 4 points, 61%–80% of skin involvement; clinical remission defined as erythema, blisters, erosions completely subsided. The patients were given methylprednisolone for venous transfusion or oral administration, and some drugs for external use to prevent infection and promote skin healing. Blood samples were obtained from patients prior to therapy as well as at regular intervals after effective therapy with symptomatic improvement and the scores were 0.

### Antibodies

The following mouse anti-human mAbs and the corresponding isotype control Abs were used for flow cytometry: PE-conjugated CD3 (eBioscience, San Diego, CA); FITC-conjugated CD4 (eBioscience); PE-Cy5-conjugated CD8 (eBioscience); PE-conjugated CD4 (eBioscience); PerCP-eFluor710-conjugated PD-1 (eBioscience); FITC-conjugated CXCR5 (R&D Systems, Minneapolis, MN); and PerCP/Cy5.5-conjugated ICOS (BioLegend, San Diego, CA).

### Serum Analysis

Serum IL-21 levels and anti-BP180-NC16A titers were measured using enzyme-linked immunosorbent assays (ELISAs) (Senxiong Biotech, Shanghai, China for IL-21; MBL, Nagoya, Japan for anti-BP180-NC16A) according to the manufacturers’ instructions. Optical densities were measured at 450 nm for IL-21 and anti-BP180-NC16A.

### Cell Isolation and Flow Cytometry

Blood samples were collected into tubes containing EDTA, and PBMCs were isolated from whole blood using a lymphocyte-separating solution (Institute of Biomedical Engineering, Tianjin, China). PBMCs were resuspended at 2×10^6^ cells·mL^−1^ in RPMI 1640 containing 10% heat-inactivated FBS (Solarbio, Beijing, China). The PBMCs were cultured overnight in six-well plates at 37°C and 5% CO_2_. The PBMCs were centrifuged for 10 min and resuspended in 100 µL of confining liquid containing 90% PBS and 10% FBS. The cells were then incubated for 50 min with mAbs at 4°C and washed twice with staining buffer. The cells were then resuspended in 400 µL of PBS and assayed using flow cytometry (Beckman Coulter, California, USA). Viable lymphocytes were chosen using a two-dimensional scatter plot chart composed of forward scatter (FSC) and side scatter (SSC). The percentages of CXCR5^+^PD-1^+^ and CXCR5^+^ICOS^+^ cells were determined among CD4^+^ T lymphocytes. The cells were then analyzed using the supporting flow cytometry software.

### CD4+T cells/B cells Co-cultures

Peripheral CD19^+^ B cells and CD4^+^ T cells were isolated from PBMC of BP patients by using the Magcellect human cell isolation kit following the manufacturer’s instructions (R&D Systems, Minneapolis, MN). Purity of the recovered CD4^+^ T and B cell subsets were >95%. A total of 1.5×10^5^ CD4^+^ T cells and 1.5×10^5^ purified CD19^+^ B cells of healthy control and BP patient were cultured alone or together in triplicate in 200 µl on the anti-CD3-precoated microtiter wells. The secretion of anti-BP180-NC16A Ab in supernatant were determined by anti-BP180-NC16A EILSA kit after 7 days and each experiment was repeated three times. To evaluate the function of Tfh cells in BP autoantibody secretion, CD4^+^CXCR5^+^ T cells were cleaved from the CD4^+^T cells/B cells co-cultures system by sorting on a FACSAria flow cytometer (BD Biosciences, San Jose, CA). In some experiments, the effect of inhibiting IL-21 were examined by adding anti-IL-21 neutralizing polyclonal Ab (1 µg/mL)(LifeSpan Bio, Seattle, WA) in the T cells/B cells co-culture system.

### Statistical Analysis

The data were analyzed using the SPSS 12.0 statistical software. The Wilcoxon rank sum test was chosen for two independent samples, whereas the Wilcoxon signed-rank test was used for two related samples. Spearman’s rank correlation was used for correlation analyses. Differences with *P*<0.05 were considered statistically significant. GraphPad Prism 5 was used to complete the figures.

## Supporting Information

Table S1 **Patient characteristics**
(DOCX)
