# Supplementary material for: Follicular Helper T Cells (Tfh) and IL-21 Involvement in the Pathogenesis of Bullous Pemphigoid
Source: PLoS One. 2013 Jul 5;8(7):e68145. doi: 10.1371/journal.pone.0068145 (PMC3702561; doi:10.1371/journal.pone.0068145)
Supplement: Table S1 — Patient characteristics (DOCX) [file pone.0068145.s001.docx]

**Table S1 Patient characteristics**

| ***Number*** | ***Sex*** | ***Age(years)*** | ***Anti-BP180-NC16A***  ***(index value)*** |
| --- | --- | --- | --- |
| BP01 | F | 70 | 190 |
| BP02 | M | 55 | 97 |
| BP03 | M | 72 | 160 |
| BP04 | F | 63 | 109 |
| BP05 | M | 66 | 167 |
| BP06 | M | 65 | 224 |
| BP07 | F | 54 | 151 |
| BP08 | F | 77 | 51 |
| BP09 | M | 52 | 111 |
| BP10 | F | 56 | 66 |
| BP11 | M | 70 | 34 |
| BP12 | F | 45 | 130 |
| BP13 | M | 76 | 132 |
| BP14 | F | 66 | 176 |
| BP15 | M | 89 | 230 |
| BP16 | M | 55 | 29 |
| BP17 | M | 78 | 214 |
| BP18 | M | 85 | 125 |
| BP19 | F | 40 | 154 |
| BP20 | F | 32 | 145 |
| BP21 | M | 79 | 140 |
| BP22 | M | 67 | 150 |
| BP23 | M | 67 | 150 |
| BP24 | M | 47 | 87 |
| BP25 | M | 51 | 129 |
| BP26 | M | 78 | 158 |
| BP27 | M | 65 | 132 |
| BP28 | M | 56 | 151 |
| BP29 | F | 49 | 214 |
| BP30 | F | 44 | 187 |
| BP31 | M | 80 | 136 |
| BP32 | M | 79 | 216 |
